# Melanotic neuroectodermal tumor of infancy: A case report and review of the surgical treatment

**DOI:** 10.3892/ol.2014.2665

**Published:** 2014-11-04

**Authors:** YINGQIU CUI, ZHE MAO, CHUNHUI LIAO

**Affiliations:** Guangzhou Women and Children’s Medical Center, Guangzhou Medical University, Guangzhou, Guangdong 510120, P.R. China

**Keywords:** melanotic neuroectodermal tumor of infancy, mandibular tumor resection

## Abstract

Melanotic neuroectodermal tumor of infancy (MNTI) is a rare, fast-growing, benign tumor originating from the neural crest. The tumor most often occurs during the first year of life. The predilection site of MNTI is the anterior maxilla, whereas lesions of the mandible are uncommon and account for only 6% of all cases. At present, the most common treatment for MNTI is surgical resection, however, tumor recurrence arises in 10–60% of cases. The optimal extent of surgical resection is a matter of debate; rapid growth and the possibilities of malignant transformation and metastasis indicate aggressive surgical resection. However, extensive resection may interfere with post-operative growth and development. The procedure should therefore preserve as much of the surrounding tissue as possible. The present case study examines a rare case of right-sided mandibular MNTI in a two-month-old female. The association between the tumor and the surrounding sclerotin, affected dental germ and the condition of the inferior alveolar nerve, were observed during the operative and post-operative periods. In addition, previous cases of MNTI were reviewed to evaluate the optimal scope of surgical resection.

## Introduction

Melanotic neuroectodermal tumor of infancy (MNTI) is a rare, pediatric tumor with 447 documented cases between 2012 and the first report of the condition in 1918 (1). It is estimated that 92.8% of MNTIs occur in the head and neck, most frequently in the maxilla (68–80%), skull (10.8%), mandible (6%) and brain (4.3%) (2), without any evident gender difference. The majority of cases occur within the first year of life, with 77% discovered prior to the age of six months. In 1966, Borello and Gorlin (3) identified elevated levels of urine vanillylmandelic acid (VMA), a marker of neurogenic tumors, in MNTI patients. Accordingly, they classified MNTI as a neurogenic tumor; a definition accepted by the Word Health Organization (4). Currently, MNTI is treated primarily by surgical resection. However, the optimal extent of surgical resection remains controversial, as the post-operative recurrence rate is as high as 60%. The majority of cases of MNTI grow rapidly and are invasive to a certain extent, while a proportion undergo malignant transformation. Therefore, extended resection is often adopted as a surgical approach in the treatment of MNTI (5). However, excessive tissue resection has the potential to adversely affect infant growth and development. The present case study examines a rare case of MNTI in the right mandible and its subsequent treatment. In this study, the effects of the tumor on the surrounding sclerotin, mandible, dental germ and inferior alveolar nerve are described based on intra-operative observations and the post-operative histopathology of the resected specimens. A literature review discussing the optimal scope of surgical resection is also presented.

## Case report

### Medical history and clinical manifestations

**Figure 1:**
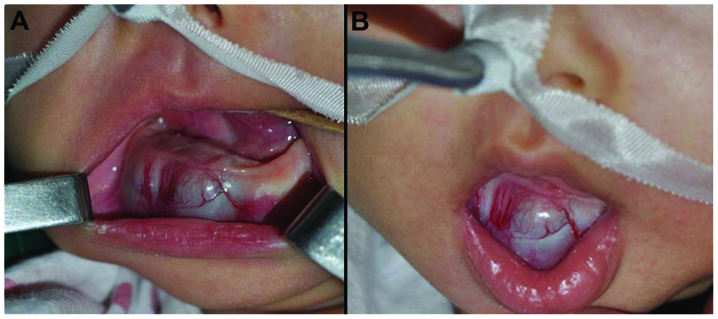
Clinical manifestations of melanotic neuroectodermal tumor of infancy originating in the mandible.

**Figure 2:**
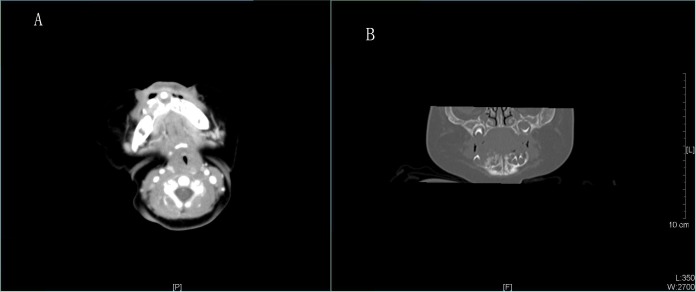
(A) Transverse and (B) coronal section computed tomography images of manibular melanotic neuroectodermal tumor of infancy of the right mandible.

**Figure 3:**
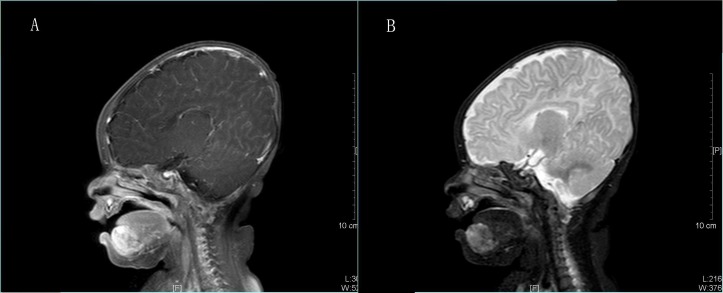
Magnetic resonance images of mandibular melanotic neuroectodermal tumour of infancy. (A) T1-weighted and (B) T2-weighted images.

A two-month-old infant female was admitted to The Affiliated Women and Children’s Medical Center of Guangzhou Medical University (Guangzhou, Guangdong, China) with a tumor on the lip side of the right inferior gum, which had appeared one month earlier. Initially, the tumor was consistent with the mucosal color of the surrounding gums, however, it became cyanotic as it increased in size. No fever or pain was reported by the infant’s parents, however, the patient was taken to hospital due to concerns that the tumor would interfere with eating and development. Upon physical examination, a projecting mass measuring 2×2.5×2.5 cm was detected on the right inferior and anterior alveolar ridge. There was no tenderness of the tumor mass, and it lay firm on the gum (Fig. 1). Upon pinpricking the right lower lip, the infant cried more noticeably compared with a healthy child. No significant anomalies were detected in the oral mucosa outside of the lesion site, and the deciduous teeth were not erupted. Computed tomography (CT) examination (Fig. 2) revealed low-density lesions of the right mandible, with unclear boundaries. On magnetic resonance imaging (MRI), T1-weighted imaging (T1WI) revealed a heterogeneous, but generally low-intensity, quasi-circular tumor (Fig. 3A). T2WI identified an uneven, higher-intensity tumor, but no invasion into the mouth floor or the vestibular sulcus mucosa (Fig. 3B). Laboratory blood tests identified no clear abnormalities in metabolism, biochemistry or coagulation functions. However, the urine VMA level was elevated at 75.9 μmol/24 h (normal range, 24.98–70.2 μmol/24 h).

### Surgical procedure

**Figure 4:**
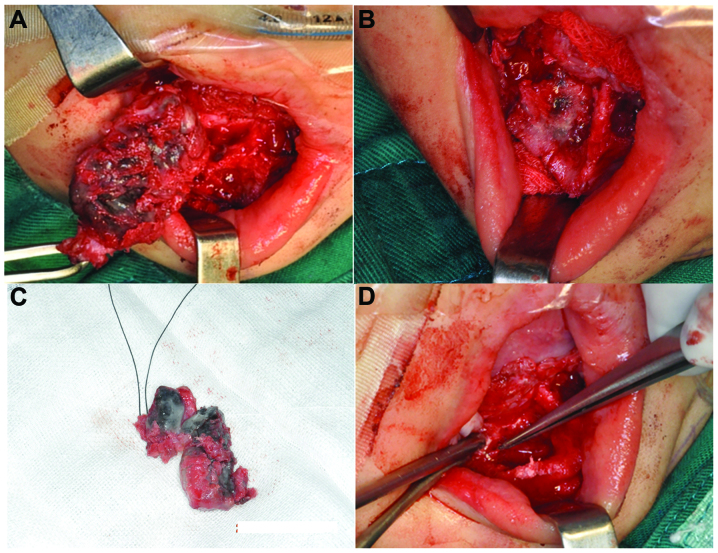
Surgical procedure and findings. (A) Tumor in the mental foramen with intact envelope. (B) Tumor exhibiting growth into the surrounding cancellous bone with visible black flecks in the sclerotin. (C) Tumor section with gray and black boundaries. (D) The remaining mandible cavity post-tumor resection.

During the surgery, the periosteum of the mandible and the inferior alveolar nerve were maintained, while tumors surrounding the sclerotin and the relevant teeth were resected. The vestibular sulcus was adopted, and the tumors were accessed following incision of the mandibular mucosa of the vestibular sulcus. The periosteum of the mandible was disrupted at the lesion sites. A solid black tumor in the mental foramen, with intact envelope (Fig. 4A), exhibited infiltrative growth into the surrounding cancellous bone (Figs. 4B and 6), and contained the dental germ of tooth 81 and 82. The tumor and its dental germ were excised for subsequent pathological examination. The remaining cavity of the mandible exhibited a honeycomb structure, and invasive black flecks were observed in the sclerotin (Fig. 4B). The sclerotin around the tumors was resected for pathological examination, and areas invaded by black flecks were removed using an abrasive drill until the remaining bone was smooth. Due to contact of the inferior alveolar neural tube with the tumor, the sclerotin around the mental nerve was removed, but the mental nerve itself was retained. Following tumor resection (Fig. 4D), the tumor envelop was found to be intact, with black and white stripes visible on the side of the section. Resection of the tumor resulted in a reduction in the thickness of the mandible cortex; in order to prevent fracture, a titanium plate was inserted to strengthen the lower edge of the mandible.

### Pathological examination

#### Tumor tissue

**Figure 5:**
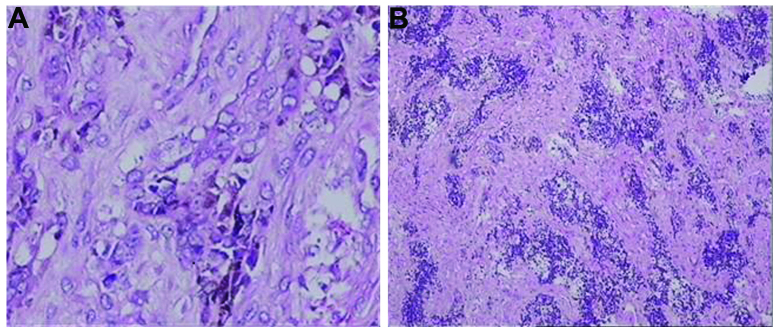
Cancer cell types of melanotic neuroectodermal tumor of infancy in the fibrous connective tissue. (A) Larger pigmented cells (magnification, 40) and (B) nested small round cells (magnification, ×400) (stain, hematoxylin and eosin).

The primary tumor volume was 3×2×1.2 cm, and two teeth were found on its surface without an obvious envelop. The side of the section side was gray and black, with clear boundaries visible (Fig. 4C). No noticeable tumor invasion was identified in the teeth. Microscopic examination revealed nested, small round cells and larger pigmented cells in the fibrous connective tissue, without evident pathological nuclear fission, necrosis or perineural invasion (Fig. 5). Smaller round cells were vimentin/neuron-specific enolase (NSE)/melanoma-associated antigen 45 (HMB45)/synaptophysin(+), and larger cells were cytokeratin (CK)/epithelial membrane antigen(+), with no significant glial fibrillary acidic protein (GFAP)/S-100 staining. Scattered cells exhibited desmin immunoreactivity, and ~2% of the cells were Ki-67(+).

#### Sclerotin around the tumors

**Figure 6:**
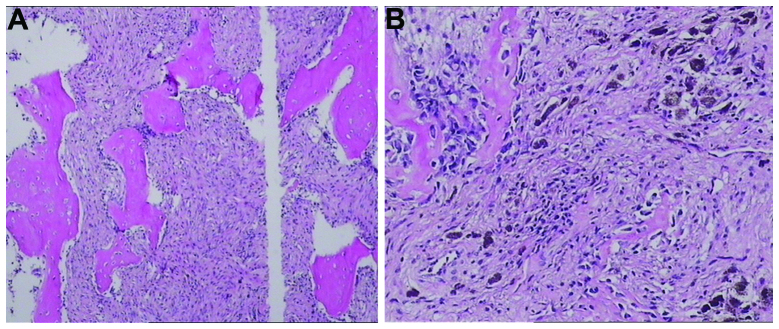
Tumor cell invasion into the surrounding sclerotin. (A) Tumor cells separate from the bone trabecula (magnification, ×400). (B) Tumor cell invasion of the bone trabecula (magnification, ×40) (stain, hematoxylin and eosin).

The mandibular tissue was disrupted by the tumor (Fig. 4C). Certain tumor cells within the sclerotin were pigmented, but without a noticeable allotype (Fig. 6).

#### Sclerotin around the mental foramen

**Figure 7:**
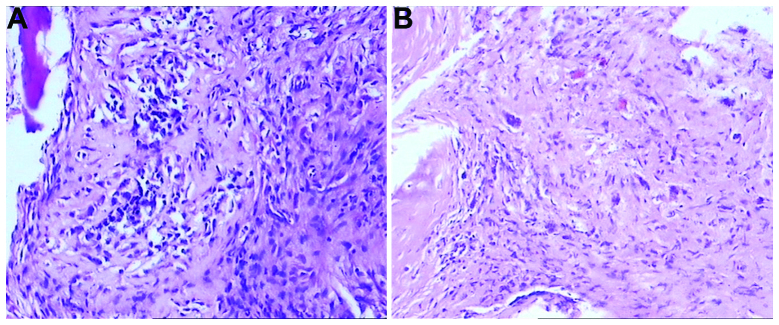
Inferior alveolar neural tube invasion by the tumor cells in the (A) soft tissue and (B) sclerotin (stain, hematoxylin and eosin; magnification, ×40).

Fewer pigmented epithelial tumor cells were present within the bone tissue (Fig. 7).

### Post-operative conditions

**Figure 8:**
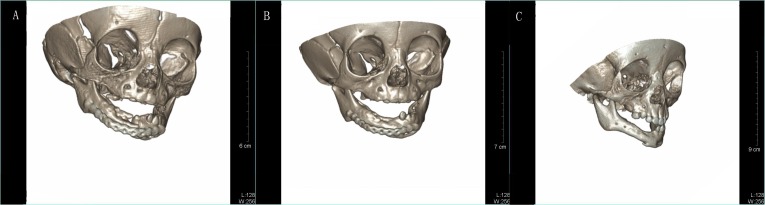
Computed tomography images acquired to observe mandible development (A) immediately after surgery, (B) four months after surgery, and (C) one year after surgery.

In order to detect any recurrence of the MNTI and observe the development of the mandible, CT images were acquired immediately after surgery, and at four months and one year post-surgery (Fig. 8). The titanium plate implanted during the surgery was removed four months later. Observational results revealed no tumor recurrence and good development of the remaining mandible.

## Discussion

MNTI most often occurs in the first year of life, although rare adult cases have been reported (6). There is a general agreement that the neural crest is the origin of MNTI for the following reasons: i) The tumor cells are similar to neuroblasts with respect to histological evaluation; ii) neurosecretory granules can be observed under an electron microscope, and iii) the catecholamine metabolite VMA level increases in the urine. Furthermore, the levels of VMA gradually return to normal following tumor resection (7). MNTI that originates from the bone is associated with osteolytic bone destruction, expansive bone destruction, cystic bone destruction, hyperosteogeny and osteosclerosis. Typical CT images reveal low-density masses with irregular edges. However, MNTI occasionally manifests as higher-density lesions with clearer boundaries between surrounding tissues. The appearance of MNTI on CT provides important information for surgical design. MRI can identify hypodense masses, with focal areas of hyperdensity in T1WI images, and isointense masses on T2WI images (8). Upon pathological examination, MNTI is usually composed of large pigmented epithelioid cells and small neuroblastoma-like cells (9). It is known that tumor cells exhibit heterogeneous immunoreactivity. There is usually a high expression level of CK and HMB-45 in the large pigmented epithelioid cells, but a low expression level of S-100 protein. In the smaller neuroblastoma-like cells, cluster of differentiation (CD)56 and synaptophysin are expressed (10). In the majority of cases, NSE can be detected in the two cell types. Certain MNTI cells may also express Ki-67/CD99 positivity, which indicates a faster growth rate (11).

On clinical examination, MNTI must be distinguished from developmental cysts, odontogenic lesions (including odontoma, enameloblastoma, odontogenic myxoma and odontogenic keratocysts), non-odontogenic and non-cancerous lesions (including eosinophilic granuloma and fibrous dysplasia), and non-odontogenic benign tumors (including rhabdomyosarcoma, Hodgkin’s lymphoma, Langerhans cell syndrome and Ewing’s sarcoma). A differential diagnosis can be made according to the typical clinical and imaging manifestations of MNTI, as well as the histopathological hallmarks, such as the epithelioid and neuroblastoma-like biphasic differentiation of tumor cells and the existence of pigment (2). However, biopsy results may be equivocal and therefore have the potential to make the differential diagnosis challenging. The epithelioid cells of MNTI bear resemblance to melanocytes, which are indicative of malignant melanoma, although malignant melanoma of the oral mucous membrane is rare in children. Therefore, a diagnosis of malignant melanoma of the oral cavity mucous membrane in children requires a more in-depth investigation. The typical immunohistochemical staining patterns of melanoma are CK(−)/HMB45(+)/S100(+), as opposed to the MNTI pattern of CK(+)/HMB45(+)/S100(−). If the tumor specimen consists mainly of neuroblastoma-like cells, with suspected malignancy upon clinical evaluation, it should be classified as a ‘small round cell’ tumor arising in infancy, such as neuroblastoma or rhabdomyosarcoma (12). Neuroblastoma cells can form rosettes, with retinal ganglial cell differentiation in certain cases. In rhabdomyosarcoma, certain cells exhibit red pigmentation in the cytoplasm, and the immunohistochemical expression pattern is desmin(+)/muscle regulatory protein MyoDl(+)/NSE(−). Conversely, the typical MNTI pattern is NSE(+)/MyoD1(−). Other small round cell tumors (such as those appearing in Ewing’s sarcoma, peripheral primitive neuroectodermal tumors and desmoplastic small round cell tumors) are rare prior to the age of five (13).

There is no typical biological behavior of MNTI. Although it is locally fast-growing and is considered benign, recent studies have indicated that the local recurrence rate following conservative resection is 10–60%, with 6.5% of cases also showing distant metastasis (14). Recurrence may occur due to invasion of the tumor edge into the bone, difficulty in complete resection due to a tumor with no envelope (15) or multicenter growth. In previous studies, high recurrence rates and no recurrence despite incomplete resection have been reported (16). The lack of recurrence without complete resection may stem from a compaction effect that triggers an immune response to destroy the remaining tumor cells. Other studies have proposed that peripheral cells depend on a group of stimulating cells in the tumor center, therefore, when the central stimulating cells are removed, peripheral tumor cells also die (17).

The primary treatment for MNTI is surgical resection, although there are examples of MNTI treatment using chemotherapy alone. However, it is generally agreed that chemotherapy is indicated for patients not amenable to surgical treatment, or for use as an adjuvant therapy prior to and following surgery (18). The optimal scope of surgical resection is a matter of debate. Radical resection may reduce the risk of relapse for a fast growing tumor, and extended resection is often applied to reduce the risk of malignant transformation (19). However, the effects of radical resection on post-operative growth and development should be taken into consideration to minimize any loss of tissue function. In cases where extensive resection will not cause severe defects, it should include the removal of adjacent tissues to reduce the likelihood of malignant transformation and metastasis. When the complete removal of lesions may incur severe defects and dysfunction in the surrounding tissues, more conservative scaling can be an effective treatment option given that further treatment can be administered during follow-up (20). MNTI occurs in the head and neck in ~92.8% of cases, and as a result, the tumor has the potential to invade areas important for nerve distribution and bone development. Therefore, the surgeon must evaluate the benefits of maintaining the important nerves and periosteum during the surgery against the probability of tumor recurrence.

In the present study, the associations between MNTI and the surrounding sclerotin, inferior alveolar nerve and relevant teeth were examined during the surgery and by post-operative histopathology. During tumor removal, the periosteum of the mandible was preserved to minimize deficits in mandibular development, and teeth were identified in the tumor that was removed. Following tumor removal, honeycomb sclerotin was visible around the tumors, which contained a large number of black spots (Fig. 4). Pathological examination of the sclerotin specimens revealed the presence of tumor cells (Fig. 5), indicating that the tumors were invasive. The tumor cells could therefore not be completely removed by a single excision, increasing the likelihood of recurrence. In order to further remove the tumor cells, abrasive drilling was applied to grind the affected sclerotin. During the grinding process, it was discovered that the affected sclerotin was associated with the inferior alveolar neural tube, and subsequent pathological examination was consistent with a neural crest origin (Fig. 7). The importance of the inferior alveolar nerve meant that is was retained, whilst the sclerotin around it was removed (Fig. 4). The remaining rudimentary sclerotin of the mandible was thin and so a titanium plate was used for structural reinforcement (Fig. 8A). Since the mandible periosteum and inferior alveolar nerve were retained, the patient was monitored for tumor recurrence. During the follow-up, it was found that preservation of the inferior alveolar nerve and the mandibular periosteum did not cause relapse; rather, the mandible with preserved periosteum developed well (Fig. 8B and C) and the inferior alveolar nerve was functional.

The treatment of MNTI relies primarily on surgical resection. During the surgery, the affected sclerotin around the tumors should be completely removed. Important nerves can be preserved where appropriate to maintain function, and as much of the periosteum should be retained to reduce any negative effect on bone development. As these benign tumors may display an invasive capacity, patients should be closely monitored for any signs of recurrence for up to one year post-surgery.
